# *Yersinia* Has a Tropism for B and T Cell Zones of Lymph Nodes That Is Independent of the Type III Secretion System

**DOI:** 10.1371/journal.ppat.0020086

**Published:** 2006-09-01

**Authors:** Joan-Miquel Balada-Llasat, Joan Mecsas

**Affiliations:** Department of Molecular Biology and Microbiology, Tufts University, Boston, Massachusetts, United States of America; University of California Berkeley, United States of America

## Abstract

Pathogenic *Yersinia* have a pronounced tropism for lymphatic tissues and harbor a virulence plasmid that encodes a type III secretion system, pTTSS, that transports Yops into host cells. Yops are critical virulence factors that prevent phagocytosis by macrophages and neutrophils and *Yersinia* mutants lacking one or more Yops are defective for survival in lymphatic tissues, liver, and gastrointestinal tract. However, here we demonstrate that *Y. pseudotuberculosis (Yptb)* mutants lacking the pTTSS survived as well as or better than wild-type (WT) *Yptb* in the mesenteric lymph nodes (MLN). Infection with pTTSS mutants caused lymphadenitis with little necrosis, whereas infection with WT *Yptb* provoked lymphadenitis with multiple necrotic suppurative foci. Gentamicin protection assays and microscopic examination of the MLN revealed that pTTSS mutants resided extracellularly adjacent to B and T lymphocytes in the cortex and paracortex. WT *Yptb* was found extracellularly adjacent to neutrophils and macrophages in necrotic areas and adjacent to B and T lymphocytes in less-inflamed areas. To determine whether lymphocytes protected pTTSS mutants from phagocytic cells, *Rag1^−/−^* mice were infected with pTTSS mutants or WT *Yptb*. pTTSS mutants but not WT, were impaired for survival in MLN of *Rag1^−/−^* mice, suggesting that lymphocyte-rich regions constitute a protective niche for pTTSS mutants. Finally, we show that invasin and the chromosomally encoded TTSS were not required for *Yptb* survival in MLN. In summary, chromosomally encoded factors are sufficient for *Yptb* replication in the cortex and paracortex of MLN; the pTTSS enables *Yersinia* to survive within phagocyte-rich areas of lymph nodes, and spread to other tissues.

## Synopsis

The pathogenic bacteria, *Yersinia,* synthesize an apparatus called a type III secretion system, which transports bacterial proteins, Yops, from the bacteria into important immune cells, such as macrophages and neutrophils. Normally, macrophages and neutrophils control bacterial infections by ingesting the bacteria; however, the Yops inactivate these immune cells, which in turn, enable *Yersinia* to replicate extracellularly and cause disease in many types of tissues. Pathogenic *Yersinia* are frequently found in lymph nodes of infected hosts, and the Yops are important for the bacteria to replicate and cause disease in lymph nodes since *Yersinia* mutants that lack Yops do not colonize lymph nodes efficiently. Surprisingly, the authors found that Yersinia pseudotuberculosis lacking the type III secretion system colonizes the mesenteric lymph nodes and survives extracellularly next to lymphocytes. However, in mice lacking lymphocytes, the type III secretion mutants did not survive although wild-type *Yersinia* did. The authors' findings reveal that other bacterial factors are sufficient for mesenteric lymph node (MLN) colonization of *Yersinia* and that lymphocytes provide a protective niche for *Yersinia* strains lacking the type III secretion system. Potentially, these avirulent mutant strains, which persist for at least 5 d in the mesenteric lymph nodes, could be used as live attenuated vaccines to protect against *Yersinia* infections, or as carriers of other antigens.

## Introduction

One hallmark of *Yersinia* infections is colonization of the lymph nodes. After subcutaneous delivery of Y. pestis by the bite of an infected flea, Y. pestis usually migrates to draining lymph nodes and causes a fulminant infection leading to severe swelling of these organs, termed buboes, and then spreads systemically [1]. Y. pestis is thought to have evolved from the enteric pathogen, *Y. pseudotuberculosis (Yptb),* about 1,500–20,000 y ago [2]. However, Y. pestis acquired two additional plasmids important for its life cycle [3]. Despite the high degree of genetic similarity between the two species [2], infections with *Yptb* in humans occur after ingestion of contaminated food or water and are generally mild and self-limiting [1]. However, a frequent clinical manifestation of *Yptb* infections is mesenteric lymphadenitis [1,4]. Infection in humans with the more distantly related *Y. enterocolitica (Ye)* [2,5] generally produces gastrointestinal pathology as well as mesenteric lymphadenitis [1,4]. Thus, although enteric *Yersinia* species and Y. pestis use different means of transmission and cause different syndromes, all three species have a pronounced tropism for lymphoid tissues [1,4].

All three pathogenic *Yersinia* species carry a highly homologous 70-Kb virulence plasmid, termed pIB1 in *Yptb,* pYV in *Ye,* and pCD1 in Y. pestis [6–8]. These plasmids are key virulence factors that encode the structural (Ysc), regulatory (Lcr), and effector (Yops) proteins, of a type III secretion system (TTSS) [9,10]. The structural genes of the pIB1-encoded TTSS (pTTSS) are primarily encoded in two operons, *yscAL* and *yscNU.* These two operons are separated by a bicistronic operon containing *virG,* which encodes a lipoprotein [11], and the *lcrF,* which encodes the transcriptional activator of the *ysc* and *yop* promoters [12,13]. The pTTSS allows *Yersinia* to secrete and translocate Yops into host cells [9]. It is thought that Yops are critical in allowing Yersinia spp. to reside extracellularly in infected tissues and to resist clearance by normal innate immune functions by a number of mechanisms, including apoptosis, down-regulation of pro-inflammatory responses to *Yersinia,* and prevention of phagocytosis [9,14–23]. For instance, YopJ triggers apoptosis in macrophages in culture and Mac1^+^ cells in the mesenteric lymph nodes (MLN) of mice [15–17,24]. In cultured cells, YopH, YopO, YopE, and YopT have all been implicated in preventing phagocytosis by macrophages and neutrophils [18–21]. Moreover, YopE, YopH, and YopJ decrease the levels of pro-inflammatory cytokines and chemokines [17,22,23,25]. Thus, Yops disrupt normal processes of cultured cells and inhibit innate host defense mechanisms in infected tissues.

The importance of Yops for the virulence of pathogenic Yersinia spp. has been studied in many different animal models and by different routes of infection [24,26–31]. In *Yptb, yop* mutants are attenuated for virulence after intragastric, intraperitoneal, or intravenous inoculation [24,28,29,32], and several *yop* mutants are defective for colonizing intestinal and lymphoid tissues of mice [24,27]. In addition to the pTTSS, *Y. pestis, Yptb,* and highly pathogenic *Ye* subspecies each have a chromosomally encoded TTSS, cTTSS [33,34]. The cTTSS in *Ye* is important for virulence [35–37], but differs considerably from the cTTSS encoded by Y. pestis and *Yptb,* and the role of the Y. pestis and *Yptb* cTTSS has not been extensively studied [33,38].

Our previous work demonstrated that *Yptb* strains lacking one or more Yops were deficient in colonization of the gastrointestinal (GI) tract, Peyer's patches (PP), and MLN [27]. In control studies, we tested the ability of strains lacking the entire pTTSS to colonize different tissues after intragastric inoculation of mice, expecting that these strains would be severely attenuated. Surprisingly, pTTSS mutants colonized the MLN as efficiently as or better than wild-type (WT), although they were defective in colonizing the GI tract, spleen, and liver. Further investigation into the behavior of WT and pTTSS mutants revealed that both were found predominantly in the cortex and paracortex of the MLN, and both remained extracellular regardless of the presence of the pTTSS. Our results indicate that chromosomally encoded factors are necessary and sufficient for *Yptb* to replicate within B and T lymphocyte zones of MLN.

## Results

### pTTSS Mutants Colonize the MLN and PP, but Not the GI Tract or Spleen

**Figure 1:**
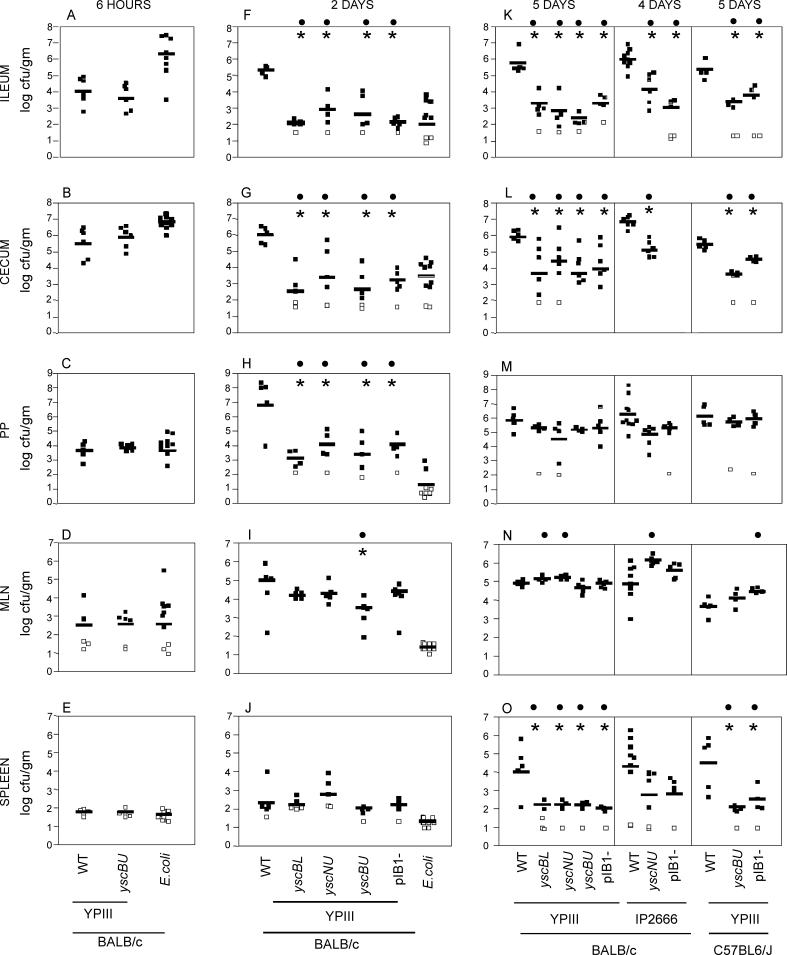
pTTSS Is Not Required for MLN Colonization BALB/c or C57BL6/J mice were intragastrically inoculated with 2 × 10^8^ WT YPIII, YPIII *yscBL,* YPIII *yscNU,* YPIII *yscBU,* or YPIII *pIB1^−^,* and colonization levels of the ileum (A), (F), and (K), cecum (B), (G), and (L), PP (C), (H), and (M), MLN (D), (I), and (N), and spleen (E), (J), and (O) were determined at 6 h (A–E), 2 d (F–J), and 5 d (K–O) post-inoculation. BALB/c mice were intragastrically inoculated with 2 × 10^8^ mouse commensal *E. coli,* and colonization levels of tissues were studied at 6 h (A–E) and 2 d post-inoculation (F–J). BALB/c mice were intragastrically inoculated with 2 × 10^9^ of WT IP2666, IP2666 *yscNU,* or IP2666 *pIB1^−,^* and colonization levels were determined at 4 d post-inoculation ( [K–O], middle section). Data are from 4–12 mice from at least two different experiments. All data was combined for each strain, tissue, and time point. Each square represents the log cfu/g tissue from one mouse; open squares indicate that less than 10 cfu were recovered per tissue, and bars represent the geometric mean. Asterisks (*) and black circles (•) indicate statistically significant differences between the WT and the mutants, calculated by the *t* test (*p < 0.01) or by Mann-Whitney (•*U* < 0.05), respectively.

To determine whether the pTTSS was required for colonization of various tissues after intragastric inoculation, several *Yptb* mutants with deletions in the operons encoding the pTTSS were created. Deletions of the *yscBL* operon, the *yscNU* operon, or both operons and the *virGlcrF* operon, designated *yscBU,* were generated. A strain lacking the pIB1 virulence plasmid was also tested to determine whether other plasmid-encoded genes were important for colonization. All four pTTSS mutants, *yscBL, yscNU, yscBU,* and *pIB1* ^−^ were used to intragastrically inoculate BALB/c mice, and their colonization levels were compared to the isogenic WT strain, YPIIIpIB1. At 6 h, 2 d, or 5 d post-inoculation, the lumen contents of the ileum, cecum, and ascending colon, the PP, cecal lymph node (CLN), MLN, and spleen were dissected, and the colony forming units per gram (cfu/g) tissue determined. At 6 h post-inoculation, no difference in colonization of any tissue was observed between the *yscBU* mutant and WT YPIIIpIB1 (Figure 1 A–1E, and unpublished data). At 2 d post-inoculation, the pTTSS mutants were severely attenuated for colonization throughout the GI tract, PP, CLN (Figure 1F–1J, and unpublished data). In contrast, the level of colonization of the MLN was similar between WT, and three of the four TTSS mutants, *yscBL, yscNU,* and *pIB1^−^* indicating that the TTSS was not required for colonization of MLN. At 5 d post-inoculation, the defect in colonization of the pTTSS mutants was evident throughout the GI tract and spleen, whereas colonization levels of all pTTSS mutants in the MLN, PP, and CLN were similar to WT or in some cases higher than WT (Figure 1K–1O and unpublished data).

To determine whether pTTSS mutants colonized other strains of mice efficiently, C57Bl6/J mice were infected with WT YPIIIpIB1, *yscBU,* or *pIB1^−^* (Figure 1K–1O). As observed in BALB/c mice, the pTTSS mutants colonized the PP and MLN at levels comparable to WT YPIII and were deficient in colonizing the ileum, cecum, and spleen. Therefore, the pTTSS or other elements encoded on the pIB1 plasmid are not required for *Yptb* survival and replication in the PP or MLN at 5 d post-inoculation in BALB/c or C57Bl6/J mice.

To confirm that the ability of *Yptb* to colonize the MLN in the absence of pTTSS was not due to a unique property of the YPIII strain, similar studies were performed using the *Yptb* strain, IP2666. IP2666, unlike YPIII, has a functional copy of PhoP which enables IP2666 *Yptb* to replicate intracellularly in bone marrow-derived macrophages [38]. Because IP2666 is slightly more virulent than YPIII during intragastric infections of mice (L. Logsdon and J. Mecsas, unpublished data), mice were sacrificed at day 4 post-inoculation (Figure 1K–1O). Surprisingly, the IP2666 pTTSS mutants, *yscNU,* and *pIB1^−^,* colonized the MLN at levels 10-fold higher than WT IP2666 (Figure 1K–1O). As observed for YPIII, the IP2666 pTTSS mutants were recovered in significantly fewer numbers in the ileum and spleen; however, about 10-fold more IP2666 pTTSS than YPIII pTTSS were recovered in the spleen, consistent with the increased virulence of the IP2666 strain (Figure 1O).

Since other Gram-negative bacteria reside in the lymphoid tissues for long periods of time [39], and commensal bacteria remain alive in dendritic cells in the MLN for several days [40], a mouse-commensal Escherichia coli strain isolated from mouse feces (see Materials and Methods) was used to intragastrically infect BALB/c mice. At 6 h post-inoculation, E. coli colonized the GI tract and lymphatic tissues at levels comparable to or higher than *Yptb* (Figure 1A–1E). However, by 2 d post-inoculation, E. coli were undetectable in the MLN and were almost cleared from the PP and the CLN (Figure 1F–1J and unpublished data). These results indicate that the ability of *Yptb* to survive in the MLN for up to 5 d is not a general property of all Gram-negative enteric bacteria. In conclusion, *Yptb* colonization of the MLN occurs in the absence of the pTTSS or other *pIB1*-encoded genes, demonstrating that chromosomally encoded factors are sufficient for MLN colonization.

### pTTSS Mutants Cause a Milder Inflammatory Response in the MLN Than WT *Yersinia*

**Figure 2:**
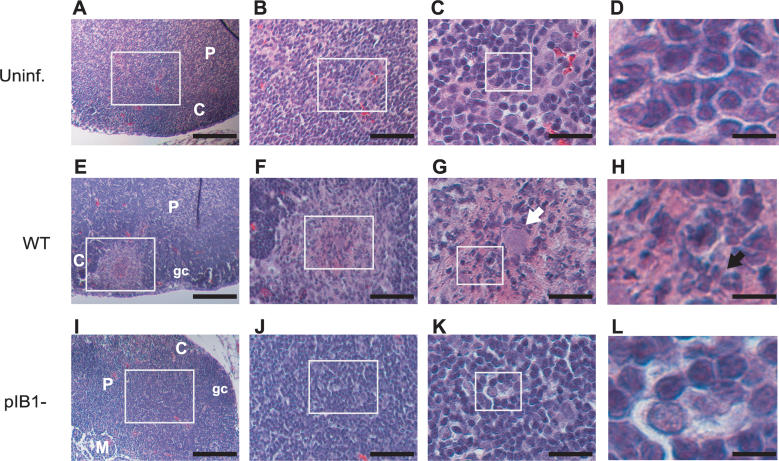
MLN Histopathology during WT or pIB1^−^ Infection BALB/c mice were intragastrically inoculated with 2 × 10^9^ WT or IP2666 pIB1^−*,*^ and MLN were processed for H&E staining 4 d later. MLN sections from uninfected (Uninf.) (A–D), WT infected (E–H), and pIB1^−^ infected (I–L) are shown at 15×, 40×, 90×, and 450× magnification. White boxes indicate magnified areas in the next slide. White arrow points to the bacterial foci, black arrow to neutrophils. Scale bars correspond to 133 μm for 15× magnification, 50 μm for 40× magnification, 22 μm for 90× magnification, and 4.4 μm for 450× magnification. Pictures shown are representative of multiple fields and samples from 16 MLN infected with WT or 14 MLN infected with pIB1^−^. C, cortex; gc, germinal center; M, medulla; P, paracortex.

To investigate the host response to infection by WT and pTTSS mutants in the MLN, infected MLN were analyzed by histology. The IP2666 strain was selected for this experiment, since the IP2666 strain was more virulent than the YPIII strain. BALB/c mice were inoculated intragastrically with 2 × 10^9^ WT IP2666 or the *pIB1^−^* mutant, and the MLN were harvested 4 d post-inoculation and prepared for histology. Gross anatomical analysis of the MLN showed that both strains caused enlargement of the MLN, i.e., hyperplasia, compared to uninfected MLN (Table 1 and Figure 2). Histological analysis of uninfected MLN showed normal follicular lymphocytic areas (Figure 2A) adjacent to a compact paracortex (Figure 2C and 2D) with no evidence of necrotic lesions or lymphoid hyperplasia (Figure 2A–2D). In most mice infected with WT IP2666, one or more MLN had multiple necrotic foci located at the edge of the MLN and close to the germinal centers (Table 1 and Figure 2E) or in the paracortex (Table 1). Areas of necrosis contained bacteria (Figure 2G, arrow), neutrophils, eosinophilic amorphous material, and many apoptotic nuclei (Figure 2H). These findings contrasted notably with MLN from mice infected with the *pIB1^−^* mutant, which had lymphoid hyperplasia and occasionally a necrotic cell but no evidence of acute necrosis (Table 1 and Figure 2I–2L). Although WT-infected MLN presented with neutrophil infiltration primarily around the necrotic foci, the *pIB1* ^−^-infected MLN generally presented with diffused infiltration of neutrophils in the paracortex. Despite the fact that IP2666 pTTSS mutants were recovered at 10 times the level of WT IP2666 (Figure 1N), no extensive necrosis was observed, suggesting that one or more components of the pTTSS trigger an acute inflammatory response leading to necrosis, whereas pTTSS mutants persist, causing milder inflammatory response.

**Table 1:**
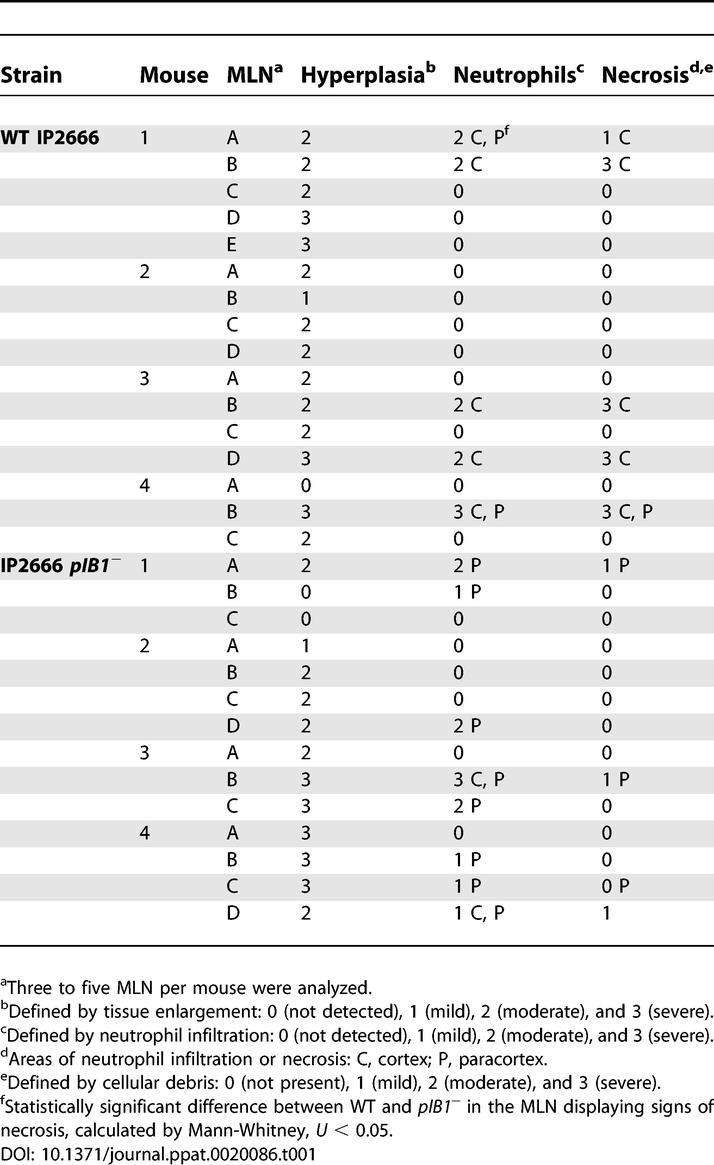
MLN Histopathological Analysis

### The *pIB1* ^−^ Strain Localizes in Lymphocyte-Rich Areas

**Figure 3:**
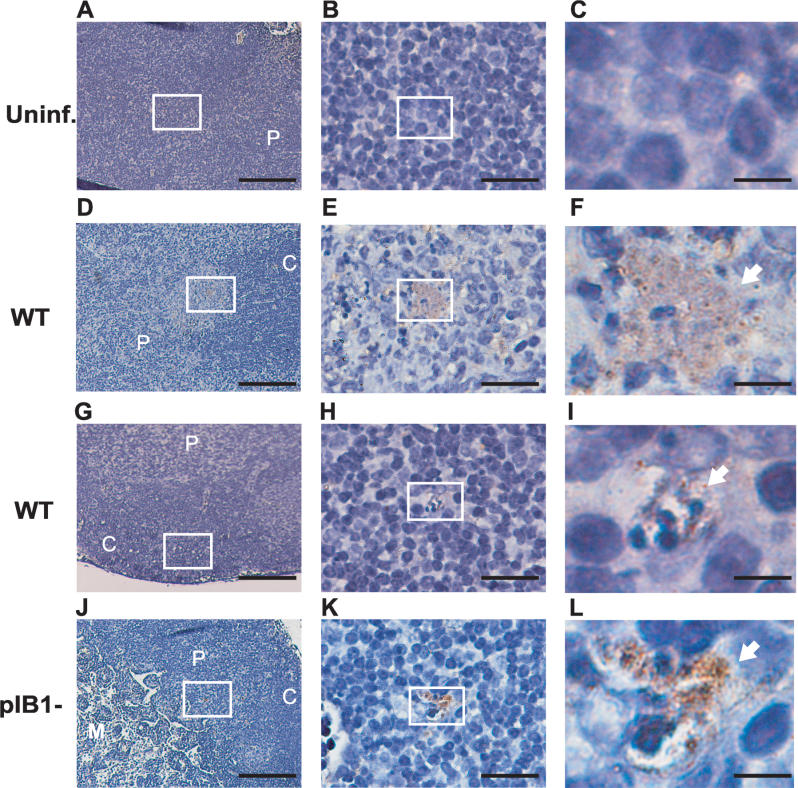
*Yptb* Localizes in the Cortex and Paracortex of the MLN BALB/c mice were intragastrically inoculated with 2 × 10^9^ WT or IP2666 pIB1^−^. At 4 d post-inoculation, MLN were harvested and stained for *Yptb* followed by hematoxylin staining. Picture sections of uninfected (A–C), WT infected (D–I), and pIB1^−^ infected (J–L) MLN were taken at 150×, 900×, and 4,500× magnification. White boxes indicate magnified areas in the next slide. Arrows point to *Yptb* microcolonies. Scale bars correspond to 133 μm for 15× magnification, 22 μm for 90× magnification, and 4.4 μm for 450× magnification. Pictures shown are representative of multiple fields and samples from MLN infected with WT or pIB1^−^. C, cortex; gc, germinal center; M, medulla; P, paracortex.

Immunohistochemistry studies were undertaken to determine the microenvironment of WT and pTTSS mutants in the MLN. WT IP2666 microcolonies were found in necrotic areas (Figure 3), in lymphocyte-rich areas of the cortex and paracortex that had no evidence of acute necrosis although apoptotic nuclei were often observed close to the bacteria (Figure 3G–3I), and in the medulla (unpublished data). *pIB1* ^−^ microcolonies were found in lymphocyte areas of the cortex and paracortex, but not in the medulla (Figure 3J–3L and unpublished data). In the cases in which infiltration of phagocytes were observed, *pIB1^−^* mutants were not detected in these areas (unpublished data), unlike in necrotic areas containing WT bacteria.

**Figure 4:**
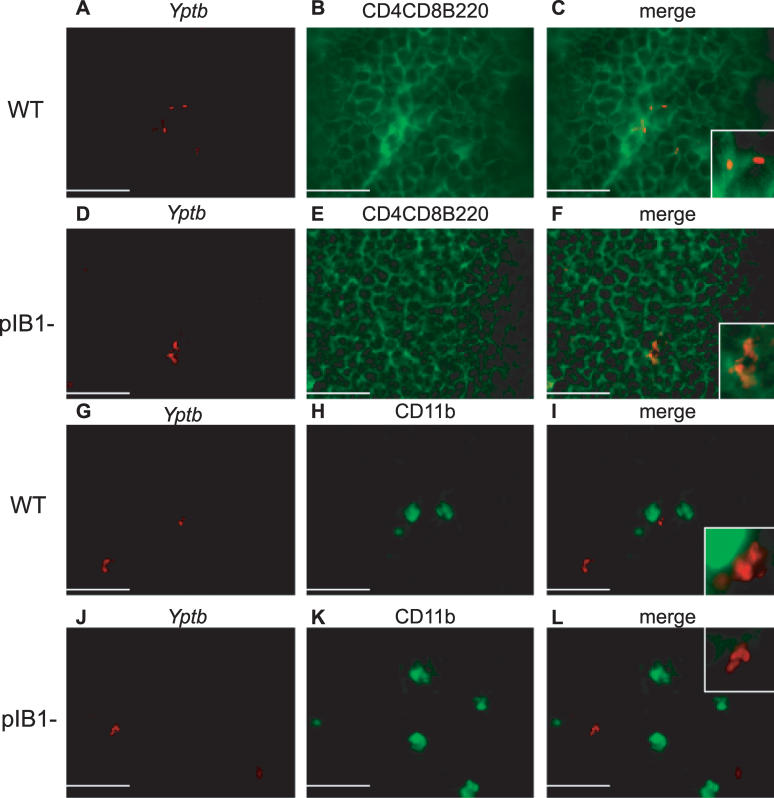
pTTSS Mutants Are Adjacent to B and T Lymphocytes Whereas WT Is Adjacent to B and T Lymphocytes and CD11b^+^ Cells BALB/c mice were intragastrically inoculated with 2 × 10^9^ WT IP2666 or IP2666 pIB1^−^. At 4 d post-inoculation, MLN were harvested, sectioned, and examined by fluorescence microscopy. Staining with antibodies to *Yptb* (red) (A), (D), (G), and (J), to CD4-CD8-B220 (green) (B) and (E), or to CD11b (green) (H) and (K) was performed, and images merged (C), (F), (I), and (L). Pictures show the cortex–paracortex and are representative of multiple fields and samples for WT-infected mice and pIB1 mice stained with CD4-CD8-B220. Fewer fields had pIB1 bacteria and CD11b+ cells. Scale bars correspond to 22 μm for 900× magnification.

The cortex and paracortex are primarily composed of lymphocytes [41,42]. In order to confirm the cell types adjacent to *Yptb,* immunofluorescence studies were performed. Serial MLN sections from infected BALB/c mice were stained with anti-*Yptb* and either a mixture of antibodies that recognize lymphocytes (CD4, CD8, and B220) (Figure 4A–4F) or an antibody that recognized macrophages and neutrophils (CD11b) (Figure 4G–4L). *Yptb* pTTSS mutants were found in lymphocyte-rich areas whereas WT *Yptb* was occasionally observed adjacent to CD11b^+^ cells (Figure 4G–4L). To determine whether *Yptb* had a preference for either B or T cells, sections were stained with anti-CD4 and anti-CD8 or anti-B220 and antibody against *Yptb.* Both WT and pTTSS mutant strains were found in close proximity to both T and B lymphocytes (unpublished data), indicating no overt preference for either type of lymphocyte.

### pTTSS Mutants Are Deficient in Colonization of Lymphoid Tissues in *Rag1^−/−^* Mice

**Figure 5:**
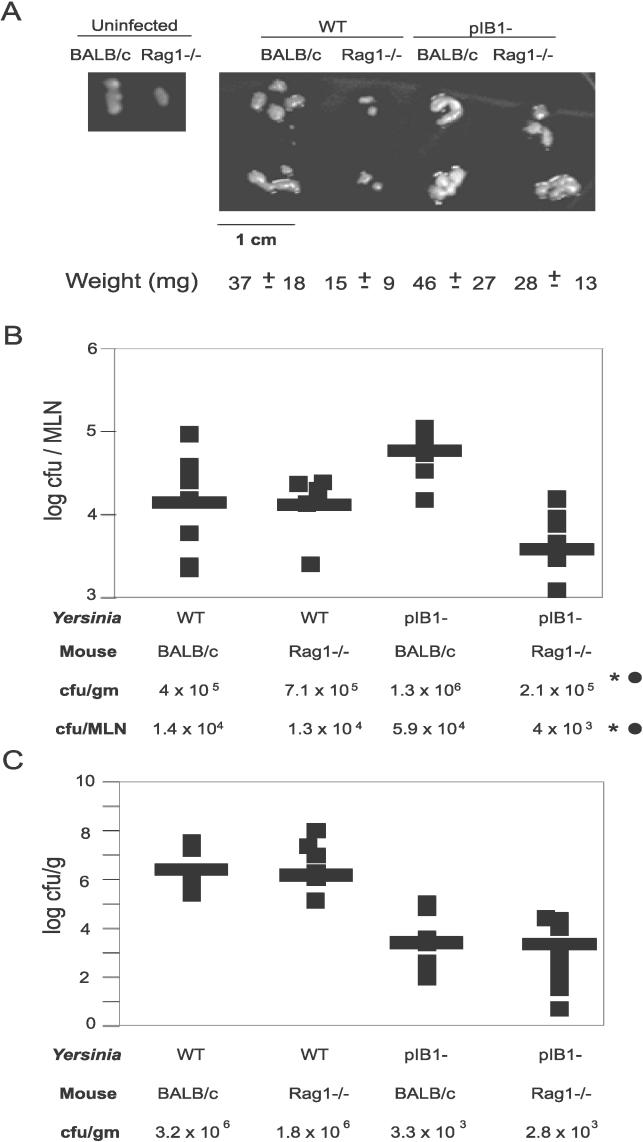
B and T Lymphocytes Are Important for pIB1^−^ Colonization in the MLN BALB/c or *Rag1^−/−^* mice were intragastrically inoculated with 2 × 10^9^ WT IP2666 or IP2666 pIB1^−^. (A) Size and weight of MLN from BALB/c or *Rag1^−/−^* mice that were either uninfected or infected with WT IP2666 or IP2666 pIB1^−^ were measured. (B) and (C) Colonization of the MLN or luminal content of the ileum 4 d post-intragastric inoculation of BALB/c or isogenic *Rag1^−/−^* mice was determined. Each square indicates the cfu from one mouse calculated as log cfu/MLN (B) or the log cfu/g of luminal content of the ileum (C); bars represent the geometric mean. Each experiment was performed with two to three mice and repeated three times. Asterisks (*) and black circles (•) indicate statistically significant differences between the number of pIB1^−^ recovered from BALB/c mice versus *Rag1^−/−^,* calculated by the *t* test (*p < 0.05) or by Mann-Whitney (•*U* < 0.05), respectively.

Since pTTSS mutants survive in B and T lymphocyte areas of the MLN, we hypothesized that B and T cell–rich areas might confer a protective niche from neutrophils and macrophages. To determine whether the absence of B and T cells rendered the IP2666 *pIB1^−^* less capable of surviving in the MLN, BALB/c or congenic *Rag1**^−/−^*** mice were infected with WT IP2666 or IP2666 *pIB1^−^*. *Rag1**^−/−^*** mice lack mature B and T lymphocytes, although small numbers of immature lymphocytes are present [43]. Four days post-inoculation, the MLN and ileum colonization levels were compared in the BALB/c and *Rag1**^−/−^*** mice (Figure 5). Notably, the size and weight of the MLN in *Rag1**^−/−^*** mice varied depending on whether mice had been infected with WT IP2666 or *pIB1^−^* strains (Figure 5A). *Rag1**^−/−^*** mice infected with pTTSS mutants had larger MLN than *Rag1**^−/−^*** MLN infected with WT *Yptb* (Figure 5A), consistent with the idea that WT IP2666 can inhibit the pro-inflammatory response [22,23,25]. Therefore recovery of bacteria in the MLN was calculated both as cfu/g and cfu/MLN (Figure 5B). Total colonization levels in the MLN were similar in WT IP2666–infected BALB/c and *Rag1**^−/−^*** mice (Figure 5B). In contrast, colonization of the MLN by IP2666 *pIB1^−^* in *Rag1^−/−^* mice was 16-fold lower than colonization levels in BALB/c mice (Figure 5B). Levels of the IP2666 *pIB1^−^* in the ileum of BALB/c and *Rag1**^−/−^*** mice showed no differences (Figure 5C), indicating that the lack of recovery in the MLN of the pTTSS mutant in *Rag1^−/−^* mice was not secondary to a reduced ability to colonize the GI tract in *Rag1^−/−^* mice.

Since *Rag1^−/−^* mice lack PP, it was conceivable that the *pIB1^−^* mutants colonize the MLN poorly because they are unable to reach the MLN due to the lack of PP, rather than to survive in the MLN. Thus, *Rag1^−/−^* mice were infected intraperitoneally (i.p.) to bypass any requirement for trafficking from the GI tract through the PP to the MLN. Unexpectedly, after intraperitoneal inoculation with either WT or pTTSS mutants, the MLN were not detectable by eye in *Rag1^−/−^* mice. Our inability to detect MLN could have been a result of their initial small size and lack of inflammatory response after i.p. inoculation, or alternatively, the MLN in the *Rag1^−/−^* mice may have been destroyed by a robust immune response to infection prior to dissection.

### pTTSS Mutants Localize Extracellularly in the MLN

**Figure 6:**
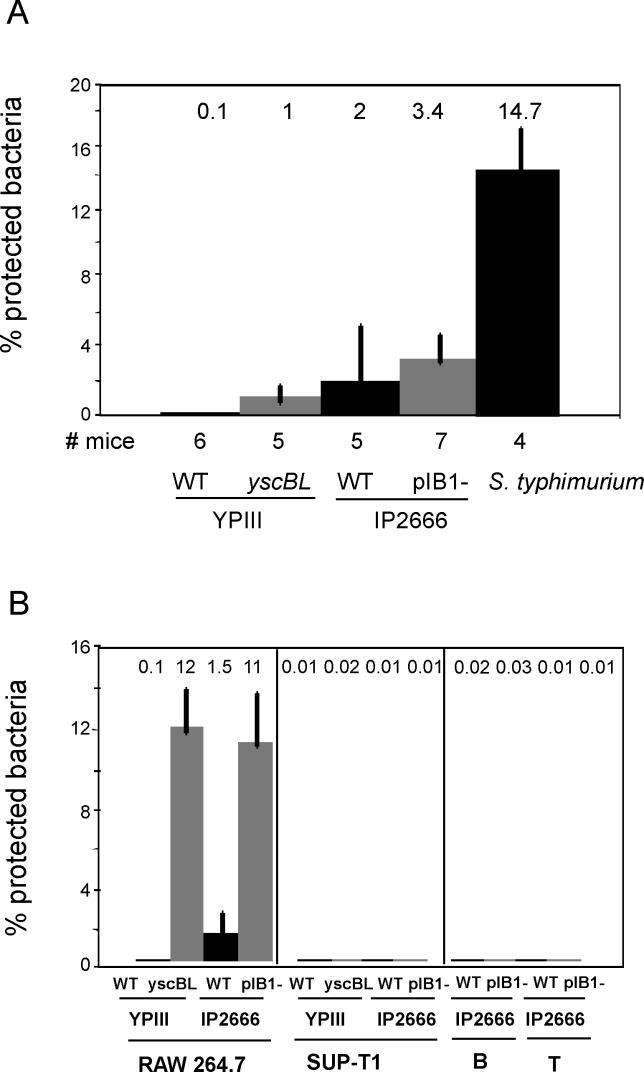
*Yptb* pTTSS Mutants Remain Extracellular in the MLN and Are Not Efficiently Internalized by B or T Lymphocytes in Culture (A) Single-cell suspensions of *Yptb*-infected MLN were assayed for the presence of intracellular bacteria using a gentamicin protection assay. BALB/c mice were intragastrically inoculated with WT YPIII (*n* = 6 mice), YPIII *yscBL* (*n* = 5 mice), WT IP2666 (*n* = 5 mice), IP2666 *pIB1^−^* (*n* = 7 mice), or S. typhimurium (*n* = 4 mice). Four days post-inoculation with IP2666 strains or 5 d post-inoculation with YPIII strains, the percentage of intracellular bacteria was assessed by generating a single-cell suspension of the MLN and treating half the suspension with gentamicin and half without gentamicin (see Materials and Methods). (B) Murine macrophage RAW264.7 cells, human T cells SUP-T1, and B and T lymphocytes isolated from MLN (see Materials and Methods) were infected with WT YPIII, YPIII *yscBL,* WT IP2666, or IP2666 pIB1^−^ strains at MOI of 10:1 for 30 min, and then treated with gentamicin for 90 min. The data are presented as 100 times the number of gentamicin-resistant bacteria divided by the number of input bacteria. Data from one representative experiment done in triplicate is shown. All experiments were performed at least three times.

Since it is well documented that the pTTSS allows Yersinia spp. to remain extracellular while docked to phagocytic cells [9], we investigated whether the pTTSS mutants were extracellular or intracellular in the MLN using an “ex vivo” gentamicin protection assay (see Materials and Methods). BALB/c mice were infected intragastrically with WT YPIII, WT IP2666, or pTTSS mutants, and 4- or 5-d post-inoculation cell suspensions of MLN were treated with gentamicin (Figure 6A). Although the differences were not significant, more pTTSS mutants were protected from gentamicin than the isogenic WT strains. However, only 1% of the YPIII *yscBL* and 3.4% of the IP2666 *pIB1^−^* mutant were protected, indicating that over 96% of the pTTSS mutants were extracellular. The observation that more WT IP2666 were protected from gentamicin than WT YPIII may reflect the ability of the IP2666 strain to survive and replicate within macrophages [38]. As a positive control, nearly 15 % of Salmonella typhimurium were intracellular in the MLN at 4 d post-inoculation. Overall, these results indicate that the majority of *Yptb* remain extracellular in the MLN, even in the absence of a functional pTTSS.

The pTTSS is important for *Yptb* to inhibit phagocytosis by macrophages, neutrophils, and epithelial cells in cell culture assays [9]; thus, it was surprising that the vast majority of the pTTSS mutants remained localized extracellularly. To confirm that the pTTSS mutants were phagocytosed more efficiently than WT, gentamicin protection assays were performed using the murine macrophage-like cell line, RAW264.7. As expected, higher level of uptake by the macrophages of the pTTSS mutants was observed compared to WT YPIII or WT IP2666 (Figure 6B). WT IP2666 was phagocytosed more efficiently than YPIII, an observation consistent with our “ex vivo” gentamicin protection assays and previous results by others [38]. *Yptb* pTTSS mutants efficiently invade some types of non-professional phagocytes, such as HEp-2 cells, by binding to α4- or α5-β1 integrins using the bacterial protein, invasin [44,45]. Since the areas of the MLN infected by pTTSS mutants are composed primarily of lymphocytes [42], and lymphocytes express α4- and α5-β1 integrins [46,47], gentamicin protection assays were performed on two human T cell lines, SUP-T1 and H9, and primary B and T lymphocytes isolated from MLN after incubation with WT and pTTSS mutants. The pTTSS mutants were not efficiently internalized by either T cell lines or by primary B and T lymphocytes isolated from MLN (Figure 6B and unpublished data). These results are consistent with the “ex vivo” gentamicin protection assays, indicating that pTTSS mutants are extracellular in lymphocyte rich areas.

### Invasin and the cTTSS Are Not Necessary for Survival in the MLN

**Figure 7:**
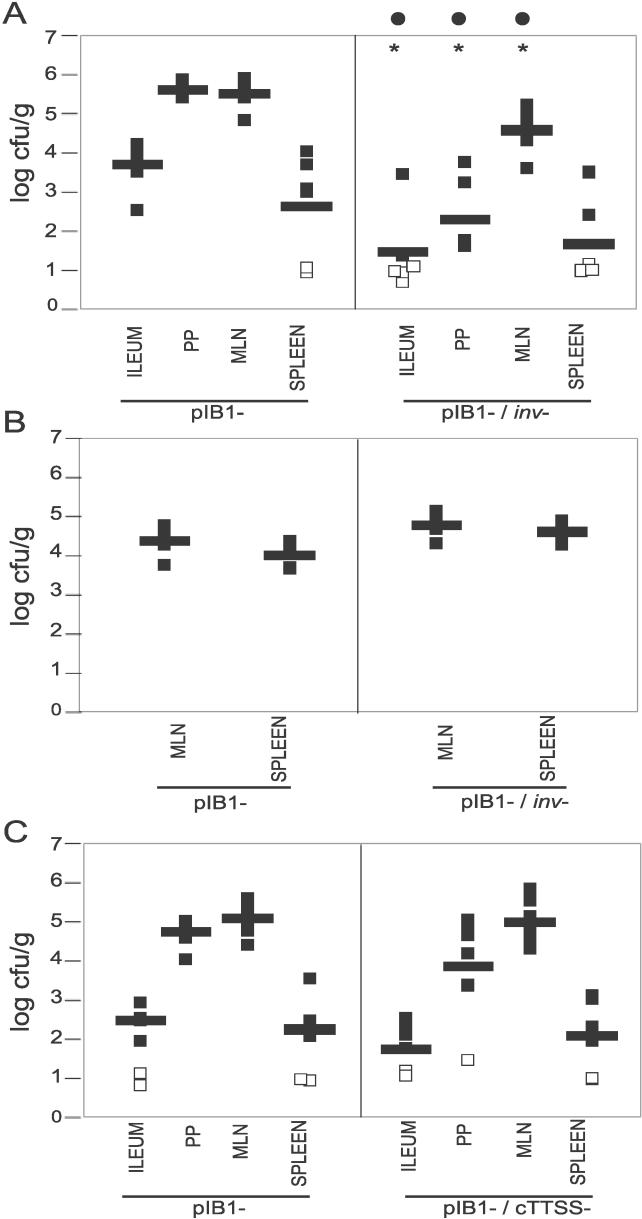
Invasin and cTTSS Are Not Essential for *Yptb* Survival in the MLN (A) BALB/c mice were intragastrically inoculated with 2 × 10^8^ YPIII pIB1 or YPIII *pIB1^−^inv^−,^* and colonization levels of the ileum, PP, MLN, and spleen were determined 5 d post-inoculation. (B) BALB/c mice were i.p. inoculated with 2 × 10^6^ YPIII pIB1 or YPIII pIB1^−^ *inv^−^,* and their colonization levels in the MLN and spleen were determined 3 d post-inoculation. (C) BALB/c mice were inoculated intragastrically with 2 × 10^8^ YPIII pIB1^−^ or YPIII pIB1^−^cTTSS, and colonization levels of the ileum, PP, MLN, and spleen were determined. Data are from 6–11 mice from at least two different experiments. Each square represents log _10_ cfu/g tissue recovered from one mouse. Bars represent the geometric mean. Open squares indicate than less than 10 cfu were recovered per tissue. Asterisks (*) and black circles (•) indicate statistically significant differences between the pIB1^−^ and either pIB1^−^ *inv* ^−^ or pIB1^−^cTTSS strains, calculated by the *t* test (*p < 0.01) or by Mann-Whitney (•*U* < 0.05), respectively.

Since chromosomal factors must play a role in *Yptb* survival in the MLN, two chromosomally encoded factors, invasin and cTTSS, were tested for growth in the MLN when the pTTSS was not present. Invasin-deficient strains *(inv^−^)* are less competent than WT in colonizing the PP following intragastric inoculation [30,48], indicating that invasin is crucial for *Yptb* to reach and/or survive in the PP. To evaluate the role of invasin in a *pIB1^−^* background, BALB/c mice were infected intragastrically and colonization levels were assessed 5 d post-inoculation. Notably, a *pIB1^−^inv* ^−^ strain was 100-fold more deficient in the colonization of the ileum compared to *pIB1^−^,* indicating that invasin plays a role in bacterial survival in the ileum that is independent of pTTSS (Figure 7A). In the PP and MLN, the *pIB1^−^inv* ^−^ strain was more deficient than *pIB1^−^* in colonization (Figure 7A); however, the lower levels in lymph tissues could reflect the lower levels of bacteria in the ileum. In order to bypass the requirement for invasin in the ileum, mice were infected intraperitoneally. No difference was observed between the *pIB1^−^* and the *pIB1^−^inv* ^−^ strains in MLN colonization after i.p. inoculation, indicating that although invasin is required for reaching the MLN after oral inoculation, it is not required for *Yptb pIB1^−^* survival in MLN (Figure 7B).

To determine whether the cTTSS is important for MLN colonization, BALB/c mice were intragastrically infected with *pIB1^−^* and *pIB1^−^cTTSS* mutants. No differences were observed in the colonization of the lumen, PP, MLN, or spleen between *pIB1^−^* and *pIB1^−^cTTSS* at 5 d post-inoculation (Figure 7C). Thus, the cTTSS is not required for MLN colonization in the absence of pIB1.

## Discussion

A prominent clinical feature of infection by pathogenic *Yersinia* is lymphadenitis, as large buboes [1] and mesenteric lymphadenitis [4] are frequent symptoms associated with infection by Y. pestis and enteric *Yersinia* pathogens, respectively. Little is known about the factors involved in *Yersinia* survival in the lymph nodes, although deletion of several Yops reduces the ability of *Yersinia* to colonize PP and MLN, which suggests that these Yops are required for reaching and/or replicating in lymph nodes [27,31]. Here, we show that *Yptb* replicates extracellularly in the cortex and paracortex of the MLN in the absence of pTTSS and Yops, indicating that the ability to reach and replicate in lymphocyte-rich areas is driven by chromosomally encoded factors. WT *Yptb* infection provoked necrotic and suppurative foci whereas pTTSS mutants induced mild lymphadenitis, demonstrating that the presence of pTTSS triggers a more acute host inflammatory response. Furthermore, B and T lymphocytes appear to provide a protective niche for pTTSS mutants since their absence results in significantly lower colonization levels of the pTTSS mutants.

Strikingly, a partial complement of Yops is more detrimental to the survival of *Yptb* in the MLN than the complete absence of pTTSS [24,27]. Strains lacking two or more Yops colonize the MLN very inefficiently [27], and strains lacking only *yopH, yopJ,* or *yopE* have a 5- to 10-fold defect in MLN colonization [24,27]. One explanation for these observations is based on the finding that the translocation apparatus damaged cultured cells and induced elevated levels of IL-8, which were decreased by YopH, YopE, or YopJ [25]. By analogy, during infection with WT or *yop* mutants, components of the pTTSS may damage lymphocytes and trigger pro-inflammatory signals in the MLN, so that in the absence of one or more Yops, the bacteria are killed by recruited macrophages and neutrophils. pTTSS mutants may survive because they do not damage host cells and therefore do not induce the same pro-inflammatory response. In addition, pTTSS mutants might replicate faster during infection than strains expressing the pTTSS, as strains lacking pTTSS have a modest growth advantage compared to WT *Yptb* in culture media (unpublished data). Nonetheless, the pTTSS mutants clearly have a growth advantage over mouse commensal E. coli in the MLN, indicating that *Yptb* chromosomal factors enable the bacteria to persist and replicate in the MLN. With this in mind, it is conceivable that a common ancestor of *Yersinia* may have been a commensal with low pathogenic capability but with the ability to persist in the MLN or host environments similar to lymph nodes of mammals. Acquisition of the pTTSS would have enabled the non-pathogenic species to spread to other tissue environments of mammals and survive within the GI tract, enhancing its transmissibility.

Although the *Yptb* pTTSS mutants are rapidly internalized by cells that are generally non-phagocytic, such as epithelial cells [30]**,** pTTSS mutants establish an extracellular niche surrounded by B and T lymphocytes in lymphoid tissues. Non-phagocytic cells expressing α4- or α5-β1 integrins internalize pTTSS mutants in an invasin-dependent manner at frequencies often 100-fold greater than WT *Yptb* [30,49]. Although T lymphocytes express α4- and α5-β1 integrins [46,47], pTTSS mutants were not internalized significantly more than WT *Yptb* during infection, and did not invade T or B lymphocytes isolated from MLN. Although lymphocytes are not generally considered intracellular hosts for bacterial pathogens, S. enterica is found within late endosomal–lysosomal compartments in B lymphocytes of the spleen after intragastric inoculation [50]. Combined, these results indicate that the cellular mechanisms exploited by *Yptb* to prompt its invasion into non-phagocytic cells are not as active in B and T lymphocytes. In addition, invasin may not be expressed in the MLN [51].

It is well established that the pTTSS is an essential virulence organelle that enables *Yptb* to remain extracellular in the presence of phagocytes through the anti-phagocytic action of Yops [9,21,52]. WT *Yptb,* but not the pTTSS mutants, was occasionally found associated with macrophages and neutrophils, suggesting that our inability to detect pTTSS mutants adjacent to CD11b^+^ cells may be due to rapid internalization and killing of these mutants by phagocytic cells. Furthermore, in *Rag1^−/−^* mice, WT *Yptb* had a selective advantage compared to the pTTSS mutants, suggesting that pTTSS mutants are compromised for survival in lymph nodes that lack B and T lymphocytes. In the absence of lymphocytes, the protective niche may be lost and the bacteria may be more exposed to phagocytes in the MLN [53]. In the PP, *Yptb* first encounters dendritic cells, neutrophils, and macrophages immediately after transcytosing the M cells [48]. In contrast to the MLN, the pTTSS mutants colonize the PP poorly at day 2, suggesting that many pTTSS mutants are eliminated by these cells prior to reaching the follicular area rich in B and T cells.

The inability of pTTSS mutants to survive well in *Rag1^−/−^* mice compared to the isogenic BALB/c mice suggests that lymphocytes provide a protective niche for pTTSS. However, it is possible that the lack of PP hindered the pTTSS in reaching the MLN. If this were true, then the lack of PP was specifically detrimental to the pTTSS mutants, since the WT *Yptb* was found in the MLN of *Rag1^−/−^* mice. The latter result indicated that WT *Yptb* does not require PP for successful dissemination to the MLN. We attempted to investigate whether the low levels of pTTSS mutants observed in the MLN of *Rag1^−/−^* mice were replicating in the MLN or were the result of continual seeding of the MLN from the GI tract. To eliminate pTTSS mutants in the GI tract and therefore prevent reseeding, mice were given streptomycin orally as described in [54]; however, in control experiments we found that streptomycin delivered orogastrically killed *Yptb* colonizing the MLN after i.p. delivery, but not *Yptb* colonizing the spleen, which is consistent with results reported about colonization of the spleen in [54]. Thus, this experimental approach is unfeasible. Future experiments are directed at determining where the pTTSS mutants are in the MLN of *Rag1^−/−^* mice, and the immune response to infection with WT and pTTSS in lymph nodes.

Common host-driven pathways indiscriminately traffic bacteria to lymph nodes, in part by dendritic cells binding to bacteria in the intestinal lumen and then migrating to the MLN [40]. Consistent with these observations, we found non-pathogenic E. coli in the MLN at 6 h post-inoculation. Also, others have reported *Yptb* and *Salmonella* in the blood as early as 30 min after intragastric inoculation [40,55]. It is noteworthy that at 6 h post-inoculation E. coli colonizes the lumen at much higher levels than *Yptb,* but their levels in the MLN are comparable to *Yptb*. Thus, *Yptb* likely use several tactics to rapidly reach the MLN, including invasin-dependent [30,48,56] and host-driven mechanisms [40,55], whereas E. coli either fails to efficiently reach the MLN or is rapidly eliminated by innate immune defenses in the MLN.

The key to the persistence of *Yptb* pTTSS mutants in the cortex and paracortex may lie in adherence factors that facilitate the interaction of *Yersinia* with lymphocytes or the underlying extracellular matrix in these areas. We have ruled out the requirement of invasin, YadA, and potential cTTSS-secreted factors in MLN colonization. Other candidate adhesion factors include the pH6 antigen [57] and glycoproteins. In other organisms, glycoproteins play a key role in conferring cellular tropism [58,59], and intriguingly, in some cases, they have been shown to be sufficient to induce a state of proliferative unresponsiveness in lymphocytes [58]. One such example is the MV glycoprotein complex of measles virus, which interferes with the propagation of the IL-2 receptor signal by blocking activation [58]. One could envision that the presence of specific glycoproteins or specific sugars on the LPS determines *Yptb* tropism and/or moderates inflammatory cytokines produced by B and T cells. In addition, it is tempting to speculate that preferential interaction with certain subsets of lymphocytes, such as CD4^+^CD25^+^ T regulatory cells may further aid *Yptb* colonization. In fact, the presence of CD4^+^CD25^+^ T lymphocytes has been shown to favor the establishment of Helicobacter pylori in the gastric mucosa by moderating inflammation [60].

Despite the key roles that the pTTSS and Yops play in disease [27], there is a report of a serotype 4a *Yptb pIB1^−^* strain isolated from a 10-y-old girl diagnosed with acute mesenteric lymphadenitis [61]. In agreement with our data, characterization of that strain in mice as well as another serotype 4a *pIB1^−^* strain demonstrated that serotype 4a strains lacking the pIB1 plasmid colonized the MLN at levels comparable to WT *Yptb* for up to 7 d [61]. Interestingly, the fate of *Ye* pTTSS mutants in the MLN seems to be dependent on *Ye* serotype. *Ye* biotype 1A *pYV^−^* strains have been isolated from symptomatic patients [62]. On the other hand, a number of experiments with other *Ye* biotypes, *Ye* O3 *pYV^−^, Ye* O9 *pYV^−^,* and Ye8081 *pYV^−^,* indicate that these strains were eliminated from the MLN of rabbits, gnonobiotic piglets, or mice within 1 to 3 d ([63–65] and unpublished data). Taken together, these results indicate that both enteric Yersinia spp. reach the MLN in the absence of pTTSS, but only a subset of strains flourishes for longer periods, and suggest that *Yptb* has specific chromosomally encoded factors that enhance its survival. A comparative analysis of the chromosomal genomes of *Yersinia* strains that persist versus those that fail to persist in the MLN may reveal factors that enable replication in lymphoid tissues.

Numerous pathogens, including Mycobacterium spp., Salmonella spp., Brucella spp., viruses, and parasites such as Leishmania spp., are capable of producing lymphadenitis [39,66–69]. These pathogens use a variety of mechanisms to survive in the MLN. For example, S. typhimurium survives intracellularly within macrophages in the MLN, where it can be isolated for up to a year post-inoculation [39]. Brucella suis survives inside macrophages by inhibiting apoptosis [70], and Leishmania spp. survive inside granulomas in the MLN [69]. We have shown that *Yptb* replicates in B and T lymphocytes areas in a pTTSS-independent manner. The presence of the pTTSS provokes many necrotic lesions, and allows *Yptb* to survive in phagocyte-rich areas cells and invade other organs. Future directions are aimed at identifying both the bacterial and host factors that permit replication of *Yptb* in lymphocyte rich zones of the MLN to understand the mechanisms driving the lymphotropism of *Yersinia*.

## Materials and Methods

### Bacterial strains.

Two virulent serotype III *Yptb* strains were used: YPIII pIB1 [27], which has recently been shown to have a mutation in *phoP* [38], and IP2666 pIBI [38]. These strains are described as WT YPIII and WT IP2666. The YPIII *pIBI^−^kan* and IP2666 *pIB1^−^* were generated by growing WT YPIII *kan* [27], or WT IP2666 on Luria Agar supplemented with 20 mM of sodium oxalate, 20 mM MgCl_2_, and 0.05 % Congo Red Dye. Loss of a functional pTTSS results in white colonies. The absence of several genes located on different regions of the plasmid was confirmed by PCR, indicating that the whole plasmid was lost. The invasin-deficient strain JM494 [30] was cured of pIB1 as described above. The mouse commensal E. coli strain was isolated from the feces of BALB/c mice (Taconic, Hudson, New York, United States). This strain was transformed with either pBR322 or pBR322-expressing *invasin,* generating JMB44 or JMB45, respectively. Expression of *invasin* in this E. coli strain increased the uptake by 100-fold in gentamicin protection assays using HeLa cells (unpublished data); however, no difference in colonization of the GI tract, PP, or MLN was observed when either E. coli strains were used to infect mice (Figure 1 and unpublished data). *S. typhimurium,* SL1344, was used [39].

Additional mutant strains generated in this study were constructed in WT YPIII or WT IP2666 by allelic exchange using the suicide vector pCVD442, and methods described in [27]. Isogenic pTTSS mutants were constructed by deleting the regions between *yscB* to *yscL, yscN* to *yscU,* and *yscB* to *yscU*. The primer sequences used to generate the appropriate suicide plasmids were as follows: *yscBL* deletion, *yscB1*-GTGTGAGTCGACGCGGTGGCGCAA GAAAC, *yscB2*-GACAGTGCATGCAGTTGGCCAACGCTTG TTGCATTAG; *yscL3*-CCAACTGCATGCACTGTCCAGGAGCTATTCGTGAAC; and *yscL4*-CTGTCAGAGCTCGTCGCTATAATTGTCTCTAC; *yscNU* deletion, *yscN1*-GTGTGAGTCGACCTACTCCCTGAGATGAAC, *yscN2*-GACAGTGCATGC AGTTGGCCAAGTGGAATAAGTAATGC, *yscU3*-CCAACTGCATGCACTGTCTCA TCAGTGGTGGTAGCT, and *yscU4*-CTGTCAGAGCTCCCAATAGCCGGTGTTAATC; and *yscBU* deletion, *yscB1, yscB2* and *yscU1, yscU2*. Colonies were screened for their ability to bind to Congo Red. Deletions were confirmed by PCR and then Southern blot.

The cTTSS deletion was constructed by deleting the putative structural genes *spiA-ssaV*. Primers used to generate the suicide plasmid were: *spiA1*-GTGTGAGTC GACCTGTGCCATAAAAGCGATCC; *spiA2*-GACAGTGCATGCAGTTGGTAG CGGAATATCGC; *ssaV3*-CCAACTGCATGCACTGTCCACATTTTGCCGTT; and *ssaV4*-CTGTCAGAGCTCCCTGTGCAATGCGGATAG. Deletions were confirmed by PCR and then Southern blot. In addition, the pTTSS mutants were grown at 26 °C and 37 °C, using Yop-inducing, i.e., calcium-depleted. medium [71], or non-inducing conditions, i.e., 2×YT supplemented with 5 mM CaCl_2_. At 26 °C, the growth of the mutants was comparable to WT. At 37 °C, the pTTSS mutants had a growth advantage compared to WT in Yop-inducing conditions, but they grew similarly when grown in non-inducing conditions. Yops were not secreted when *Yptb* were grown at 37 °C under Yop-inducing conditions [27].

The resulting mutants and strains used were given the following strain numbers: JMB35, YPIII *yscBL;* JMB31, YPIII *yscNU;* JMB38, YPIII *yscBU;* JMB5, YPIII *pIB1 kan;* JMB111, IP2666 *pIB1^−^;* JMB141, IP2666 *yscNU;* JMB100, *pIB1^−^inv* ^−^ *;* and JMB84, YPIII *pIB1^−^cTTSS*.

### Mouse infection.

Seven– to 8-w-old female BALB/c (Taconic, or Jackson Laboratories, Bar Harbor, Maine, United States), C.129S7 (B6)-*Rag1^tm1Mom^*/J, *Rag1^−/−^* (Jackson Laboratories), or C57BL6/J mice (Jackson Laboratories) were used. *Yptb* were grown and infections were performed as described in [27]. S. typhimurium and E. coli were grown in LB at 37 °C overnight with aeration. Mice were infected intragastrically with 200-μl PBS containing 2 × 10^8^ or 2 × 10^9^ *Yptb,* as indicated in the figure legends, 5 × 10^8^ *S. typhimurium,* or 2 × 10^8^ *E. coli.* Mice were infected i.p. with 200-μl PBS containing 1 × 10^6^ *Yptb*. The 6-h, 2-d, 4-d, or 5-d post-intragastric inoculation or 3-d post-intraperitoneal inoculation mice were sacrificed, and tissues were dissected and processed as described in [27]; *p*-values were determined by two-tailed, unpaired Student *t* test by comparing the log cfu/g tissue or log cfu/MLN of WT *Yptb* to each mutant.

The Institutional Animal Care and Use Committee of Tufts University approved all animal procedures.

### Histology and immunohistochemistry.

MLN from uninfected mice or mice infected intragastrically with 2 × 10^9^ *Yptb* were dissected, fixed in formalin, embedded in paraffin, and cut in 10-μm sections. Sections were treated with xylene to remove paraffin prior to staining with hematoxylin-eosin or staining for immunohistochemistry. Lauren Richey, DVM, PhD, Diplomat ACVP, and members of our lab scored MLN histology sections blindly.

Immunohistochemistry was performed by treating the sections with 3% hydrogen peroxide (DAKO, Glostrup, Denmark) for 5 min, washing with 0.05% Tween-20 in PBS (PBS-Tw) for 5 min, incubating with 1:100 rabbit anti-*Yptb* for 30 min, washing with PBS-Tw, incubating with 1:300 polyclonal goat α-rabbit Ig (DAKO) for 30 min (gift from R. Isberg), washing with PBS-Tw, incubating with streptavidin-HRP (DAKO) for 15 min, and then incubating with substrate-chromatogen solution (DAKO). MLN were counterstained with 0.5% hematoxylin and examined with a Nikon Eclipse TE2000-U microscope (Melville, New York, United States).

### Immunofluorescence.

MLN from uninfected mice or mice infected intragastrically with 2 × 10^9^ with *Yptb* were harvested and snap frozen in Optimal Cutting Temperature Compound (Tissue-Tek, Fort Washington, Pennsylvania, United States). The 10-μm sections were cut, fixed with cold 100% acetone, and stored at −80 °C. For staining, sections were washed with PBS and blocked with 2% BSA for 30 min. Antibodies used were rat anti-mouse CD8 (BD Biosciences, San Jose, California, United States) and rat anti-mouse CD4 (BD Biosciences) for T lymphocytes, rat anti-mouse B220 (BD Biosciences) for B lymphocytes, rat anti-mouse CD11b (BD Biosciences) for neutrophils and macrophages, and rabbit anti-*Yptb*. Primary antibodies were diluted 1:200 in 2% BSA and incubated with the samples for 3 h at room temperature. Slides were washed three times with PBS-Tw for 15 min per wash and then incubated for 1 h with FITC conjugated to donkey anti-rat (Jackson ImmunoResearch, West Grove, Pennsylvania, United States) and Texas-Red–conjugated donkey anti-rabbit antibodies (Jackson ImmunoResearch). Slides were washed three times with PBS-Tw for 15 min per wash, and a cover slip was placed over the tissue before visualization using 20×, 40× and 100× objectives with a Nikon Eclipse TE2000-U microscope with fluorescence. Pictures were taken using a Roper Scientific camera (Trenton, New Jersey, United States) controlled by Open Lab software.

### In vitro gentamicin protection assay.

The macrophage cell line RAW264.7 (ATCC# CRL-2278), the T cell line SUP-T1 (ATCC# CRL-1942), and the T cell line H9 (ATCC# HTB-176) were grown as recommended by ATCC (Manassas, Virginia, United States). Primary B and T lymphocytes were isolated from MLN using enrichment columns (Stem cells; R&D System, Minneapolis, Minnesota, United States). *Yptb* strains were grown under Yop-inducing conditions [71]. A total of 5 × 10^4^ or 2 × 10^5^ cells were infected at a multiplicity of infection (MOI) of 10:1 for 30 min at 37 °C in the presence of 5% CO_2_. Gentamicin was added to a final concentration of 100 μg/ml for 90 min, and then cells were washed three times with PBS. T and B cells were centrifuged at 200 g for 5 min between each wash. Cells were lysed with 100 μl of 1% Triton-X-100 for 5 min followed by addition of 900-μl LB, and lysates were diluted and plated to determine the number of intracellular bacteria. Results are presented as percent uptake equal to 100 times the recovered cfu divided by the input cfu.

### Ex vivo gentamicin protection assay.

MLN were dissected from 7–8-wk female BALB/c mice infected with IP2666 or *S. typhimurium.* MLN were placed in 1-ml RPMI on ice and then pressed through a 70-μm filter (BD Labware, Palo Alto, California, United States) with the rubber end of a 1-ml syringe plunger. The sample was divided into two 0.5-ml aliquots: one aliquot was treated with 100-μg/ml gentamicin for 2 h at 37 °C to kill extracellular bacteria, and the other aliquot was untreated for 2 h at 37 °C to calculate the total cfu recovered. Cells were washed three times with PBS and lysed with 100 μl of 1% Triton-X-100 for 5 min followed by addition of 900-μl LB, and lysates were diluted and plated to determine the number of intracellular bacteria. Results are presented as number of gentamicin resistant bacteria (recovered after 2 h incubation) divided by the input bacteria times 100.

## Abbreviations

cfu: colony forming units
CLN: cecal lymph node
cTTSS: chromosomally encoded type III secretion system
GI: gastrointestinal
i.p.: intraperitoneal/ly
MLN: mesenteric lymph nodes
PP: Peyer's patches
pTTSS: pIB1-encoded type III secretion system
TTS: type III secretion system
WT: wild-type
*Ye*: *Yersinia enterocolitica*
*Yptb*: *Yersinia pseudotuberculosis*
